# Tumour induction in BALB/c mice for imaging studies: An improved protocol

**DOI:** 10.1111/jcmm.17792

**Published:** 2023-05-29

**Authors:** Abolfazl Amini, Gholamreza Mesbah, Fatemeh Tash Shamsabadi, Mohammad Ali Zeyghami, Yaghoub Safdari

**Affiliations:** ^1^ Department of Medical Biotechnology, Faculty of Advanced Technologies in Medicine Golestan University of Medical Sciences Gorgan Iran; ^2^ AshianGanoTeb Biopharmaceutical Company Golestan University of Medical Sciences Gorgan Iran; ^3^ Department of Comparative Pathology, Urology Research Center Tehran University of Medical Sciences Tehran Iran; ^4^ Medical Cellular and Molecular Research Center Golestan University of Medical Sciences Gorgan Iran; ^5^ Department of Pharmacology, Faculty of Medicine Golestan University of Medical Sciences Gorgan Iran; ^6^ Golestan Research Center of Gastroenterology and Hepatology Golestan University of Medical Sciences Gorgan Iran

**Keywords:** cyclosporine A, immunosuppressed mice, ketoconazole, xenograft model

## Abstract

Dealing with nude mice, which lack thymus and therefore are sensitive to unsterile conditions, needs special care and laboratory conditions. For preclinical studies, especially tumour imaging purposes, in which therapeutic properties of drugs or therapeutic compounds are not studied, mice with normal immune system can be a favourable alternative if they carry tumours of interest. In the current study, we introduce an optimized protocol for induction of human tumours in BALB/c mice for preclinical studies. Immune system of BALB/c mice was suppressed by administration of cyclosporine A (CsA), ketoconazole and cyclophosphamide. The tumours of MDA‐MB‐231, A‐431 and U‐87‐MG human cancer cells were induced by subcutaneous injection of the cells to the immunosuppressed mice. Tumour size was calculated weekly. Histopathological and metastatic analyses were performed using haematoxylin and eosin staining. The combination of the three drugs was found to suppress immune system and decrease the numbers of white blood cells, including lymphocytes. At the eighth week, tumours with a dimension of approximately 1400 mm^3^ developed. Large atypical nuclei with scant cytoplasm were found to exist using histopathological analysis. No metastasis was observed in the tumour‐bearing mice. A combination of CsA, ketoconazole and cyclophosphamide can be used to suppress the immune system in BALB/c mice and induce tumours with significant size.

## 1 INTRODUCTION

Immune cells in laboratory animals with normal immune system invade tumour cells and prevent tumour growth and development, making it impossible to establish tumours with adequate size for in vivo studies. For most in vivo studies, the use of nude mice is unavoidable—where the animal immune system should be inactive to ensure that the drug (or compound) of interest can affect tumour cells. For imaging purposes, in which tumour growth suppression is not the goal of study, laboratory animals with normal immune system will be of great interest, if they carry tumours of the cells of interest. In such cases, results may be more consistent to real conditions when compared to those obtained with nude mice. Compared to nude mice, animals with normal immune system are not highly sensitive to unsterile conditions and do not need strict care during study. Considering such limitations of nude mice, numerous researchers have proposed various immunosuppression regimens for the development of drug‐induced immunosuppression.

Cyclosporine A (CsA) is an immunosuppressive peptide drug produced by the fungus *Tolypocladium inflatum*. It is a lipophilic molecule, which can readily cross the blood–brain barrier.^1^ By blocking the nuclear factor of activated T cells (NFAT), a cytokine transcription factor, CsA primarily inhibits the activation of T lymphocyte proliferation and cytokine production. Additionally, CsA affects growth and activation of B lymphocyte, regulates cell homeostasis, and prevents neutrophil and macrophage migration into the inflammatory zone. Because of these immunosuppressive effects, CsA is used to study immune response in inflammatory, autoimmune and infectious diseases.^2^, ^3^

Since 1978, CsA has been frequently used both clinically and experimentally.^4^ Many clinical researchers reported tumour xenograft development by modifying the CsA dose, administration route, combination with another drug and duration of treatment regimens.^5^, ^6^, ^7^, ^8^, ^9^ CsA is metabolized in the liver by cytochrome P450. CsA metabolism can be decelerated by cytochrome inhibitors like diltiazem or ketoconazole.^10^ The risk for opportunistic invasive fungal infections is decreased when CsA and ketoconazole are combined.^11^

In our previous work, we induced tumours of A‐431, U‐87‐MG human cancer cell tumours in BALB/c mice,^9^ using the combination of CsA, ketoconazole and cyclophosphamide. In the current study, we describe the improved protocol in detail.

## 2 MATERIALS AND METHODS

### 2.1 Reagents

Cell culture medium, Dulbecco's modified Eagle medium (DMEM), was purchased from Gibco (#11885084, USA). Similarly, foetal bovine serum (FBS) was obtained from Gibco (#26140). Trypsin (#S‐8064), and penicillin/streptomycin (Pen/Strep) (#S‐8062‐5ML) were purchased from DENAzist Asia. Trypan blue was purchased from Merck. CsA, ketoconazole, and gentamicin were purchased from Sigma‐Aldrich. Cyclophosphamide (CP) (Endoxan) was purchased from Baxter. Co‐amoxiclav (Clamox) was purchased from Daana Pharma Co.

### 2.2 Animals

BALB/c female mice weighing 20 ± 2 g (4–6 weeks old) were used in this study. The mice were initially kept in individually sized standard cages with a relative humidity of 60 ± 5% and a constant temperature of 25 ± 2°C. Access to autoclaved water and rodent food was available to all mice at all times. All procedures for anaesthesia/euthanasia and animal experiments were performed in accordance with the Institutional Animal Care and Use Committee (IACUC). Ethics approval for this study was obtained from the Golestan University of Medical Sciences (ethics registry code: IR.GOUMS.REC.1398.001).

### 2.3 Cell lines and preparation of tumour cells

All human cancer cell lines (MDA‐MB‐231 [human breast cancer], A‐431 [epidermoid carcinoma] and U‐87‐MG [human glioblastoma]) were obtained from the Pasteur Institute of Iran. The cell lines were maintained in DMEM medium supplemented with 10% FBS, 1% Pen/Strep, and 25 μg/mL gentamicin. The cells were grown in T75 flasks (SPL Life Sciences) and maintained in a standard tissue culture incubator at 37°C with 5% CO_2_. Semi‐confluent cells (MDA‐MB‐231, A‐431 or U‐87‐MG) were trypsinized by using 0.20% trypsin to detach the cells. Cells were centrifuged at 2800 rpm for 5 min, re‐suspended and washed in DMEM medium. After washing, cells were again re‐suspended in DMEM medium. The cells were counted and viability was determined by trypan blue exclusion test. A supply of live cells was kept on ice and injected immediately.

### 2.4 Immunosuppression

Healthy BALB/c female mice were divided into 10 groups (eight mice in each group). Groups 1–3 were administered 20 mg/kg CsA; Groups 4–6 were administered 30 mg/kg CsA; and Groups 7–9 were administered 40 mg/kg CsA by the intraperitoneal route on seven subsequent days. Groups 1, 4 and 7 were administered 5 mg/kg ketoconazole; Groups 2, 5, and 8 were administered 10 mg/kg ketoconazole; and Groups 3, 6 and 9 were administered 15 mg/kg ketoconazole by the oral route every day for 7 days. Groups 10 and 11 were administered 30 mg/kg CsA and 10 mg/kg ketoconazole, respectively. No treatment was given to Group 12 (control).

Autoclaved water and rodent food pellets were provided for the mice. The animals were given Co‐amoxiclav (0.1 μg/mL) by drinking water during the experiment. After completion of the study, haematology was carried out to determine total white blood cell (WBC) and lymphocyte count to confirm immunosuppression. In groups of mice with the highest levels of immunosuppression, CP was subcutaneously given at a dose of 30 mg/kg on 1 day before tumour cell injection.

### 2.5 Total WBC and lymphocyte count

Under ketamine‐xylazine anaesthesia, blood samples were taken from the submandibular vein of each animal and placed in a heparinized 1.5‐mL microcentrifuge tube. Then, total WBC and lymphocyte counts were performed in an automated haematology analyser (Sysmex XP‐300).

### 2.6 Tumour induction

In the present study, immunosuppressed female BALB/c mice were used. After shaving the body hairs of the mice, 100 μL FBS free culture medium containing cells (approximately 8 × 10^6^ either A‐431, U‐87‐MG or MDA‐MB‐231 cells) was subcutaneously administered to each mouse into the right hind flank region. Tumour growth was observed at the injection site. In order to estimate tumour volume by an external calliper, the greatest longitudinal diameter (length) and the greatest transverse diameter (width) were determined while mice were conscious. Tumour volume was calculated by the modified ellipsoidal formula: *V* = ½ (Length × Width^2^).^12^ Tumours were excised at the end of the study and used for histopathological study.

### 2.7 Histopathological analysis

Tumours were removed from the animals at the end of the study and kept in 10% neutral buffered formalin. Haematoxylin and eosin was used to stain the tumour samples, which were cut into 5 μm sections. To confirm the presence of cancerous cells, optical microscopy (Olympus) was used to observe the slices.

### 2.8 Statistical analysis

All data are presented as mean ± SD. One‐way anova followed by post hoc Bonferroni correction was applied to determine the significance of differences among groups. *p* ≤ 0.05 was considered to be significant.

## 3 RESULTS

### 3.1 Immunosuppression

**FIGURE 1:**
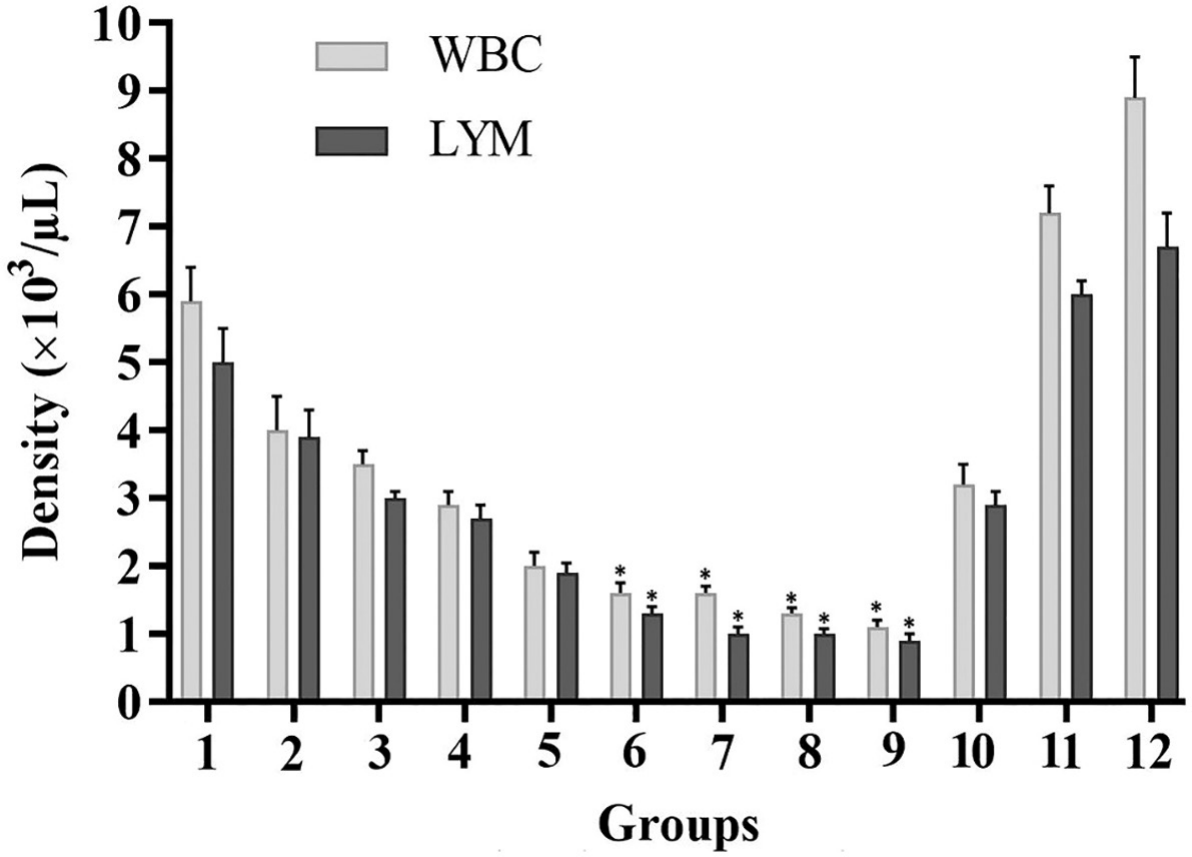
White blood cell (WBC) and lymphocyte (LYM) counts of different groups of mice following treatment with CsA and ketoconazole (*n* = 8). Groups 1–3 were administered 20 mg/kg CsA; Groups 4–6 were administered 30 mg/kg CsA; and Groups 7–9 were administered 40 mg/kg CsA by the intraperitoneal route for seven subsequent days. Groups 1, 4 and 7 were administered 5 mg/kg ketoconazole; Groups 2, 5 and 8 were administered 10 mg/kg ketoconazole; and Groups 3, 6 and 9 were administered 15 mg/kg ketoconazole by the oral route for seven subsequent days. Groups 10 and 11 were administered 30 mg/kg CsA and 10 mg/kg ketoconazole, respectively. No treatment was given to Group 12 (control). Asterisks indicate significant differences between groups (*p < 0.05).

Compared to the control mice, the combination of CsA and ketoconazole drugs significantly suppressed the immune system in BALB/c mice. Figure 1 displays the mean WBC and lymphocyte count in 12 mice groups at the end of the treatment. The results reveal an effective immunosuppression in Groups 6–9 following administration of CsA treatment (30, 40, 40 and 40 mg/kg) and ketoconazole (15, 5, 10 and 15 mg/kg), respectively. Because of more effective immunosuppression in Group 9, the mice of this group were chosen to grow tumour xenografts.

### 3.2 Tumour induction

**FIGURE 2:**
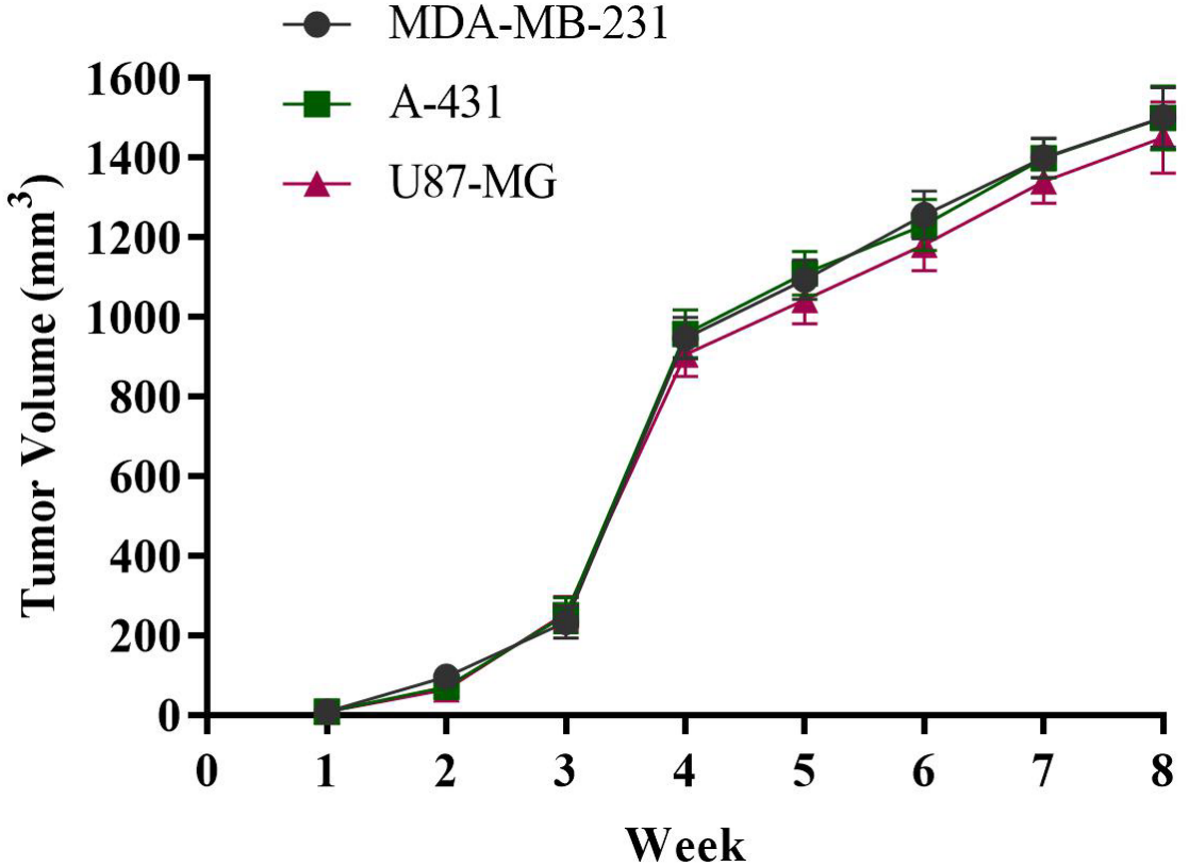
Graphs showing mean tumour volumes of MDA‐MB‐231, A‐431 and U‐87‐MG xenografts (*n* = 8).

**FIGURE 3:**
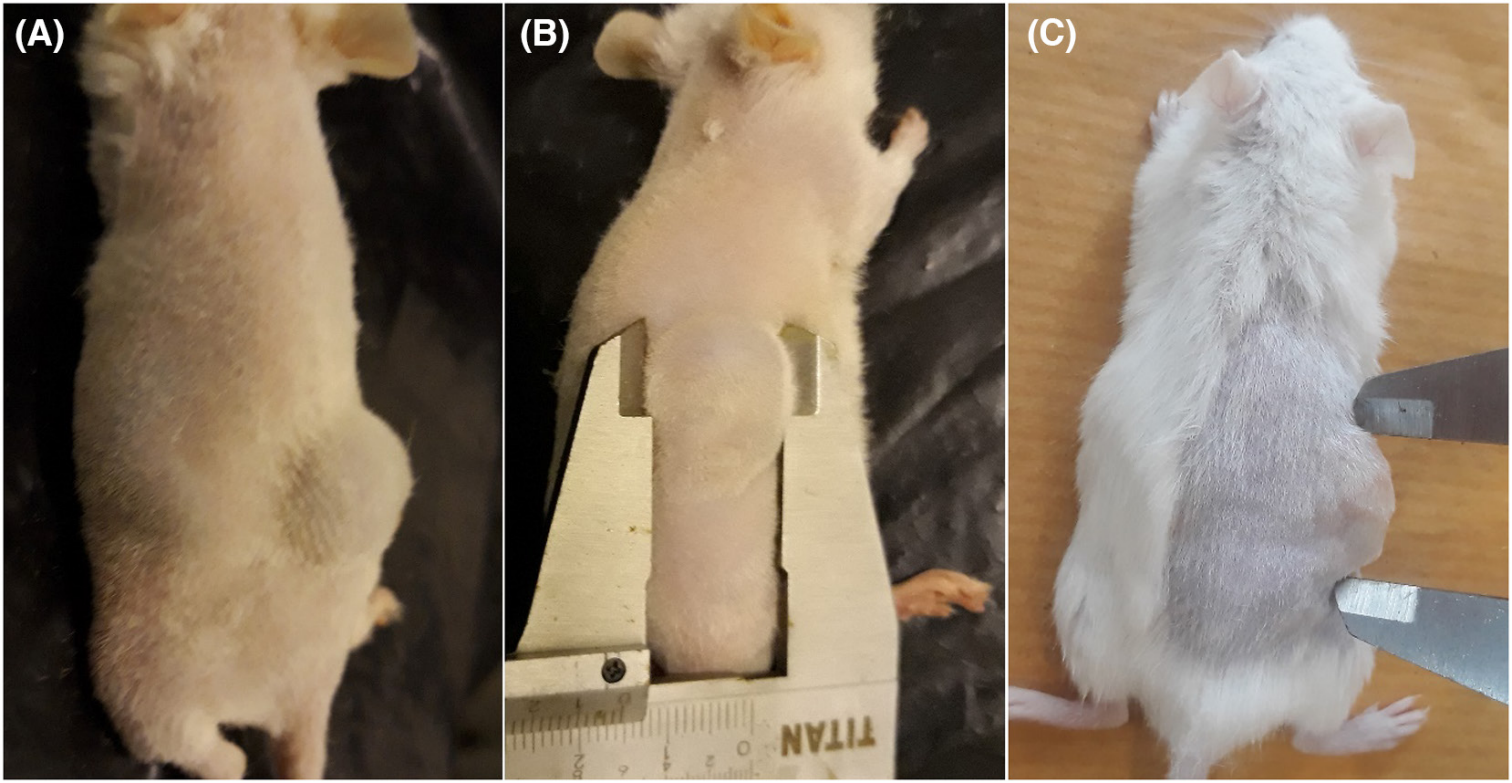
Immunosuppressed BALB/c female mice bearing tumour xenograft of (A) MDA‐MB‐231, (B) A‐431 and (C) U‐87‐MG.

Approximately 8 × 10^6^ cells from each cell line (MDA‐MB‐231, A‐431 or U‐87‐MG) were injected subcutaneously into the right hind flank region of immunocompromised mice. The mean tumour volume for each week following tumour induction is depicted in Figure 2. For each cell line, the mean tumour volume radically increased until the fourth week and then showed slow steady grow until the eighth week and finally reached a plateau. The mean tumour volumes of MDA‐MB‐231, A‐431 and U‐87‐MG xenografts were calculated to be 1502.55, 1501.2 and 1451.55 mm^3^, respectively. The tumour volume then started to shrink in some animals. Figure 3 shows BALB/c female mice bearing tumour xenograft 8 weeks after tumour induction.

### 3.3 Histopathological image analysis

**FIGURE 4:**
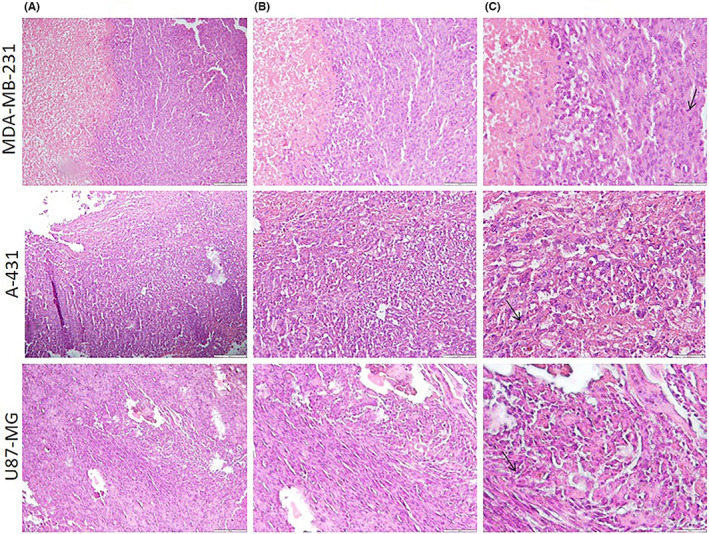
Light microscopy photomicrographs of haematoxylin‐ and eosin‐stained sections of MDA‐MB‐231, A‐431 and U‐87‐MG tumour xenograft. Light microscopy at (A) ×100 magnification, (B) ×200 magnification and (C) ×400 magnification. Mitotic figures (arrows) demonstrate neoplastic cells.

Histopathology experiments proved that a malignant tumour was present. Tumours were removed, sectioned and stained using the common haematoxylin and eosin method. Figure 4 displays slices of the MDA‐MB‐231, A‐431 and U‐87‐MG xenografts stained with haematoxylin and eosin dyes. The section displaying cells with large atypical nuclei with scant cytoplasm shows malignant cells (Figure 4c).

### 3.4 Histopathological findings of metastasis

**FIGURE 5:**
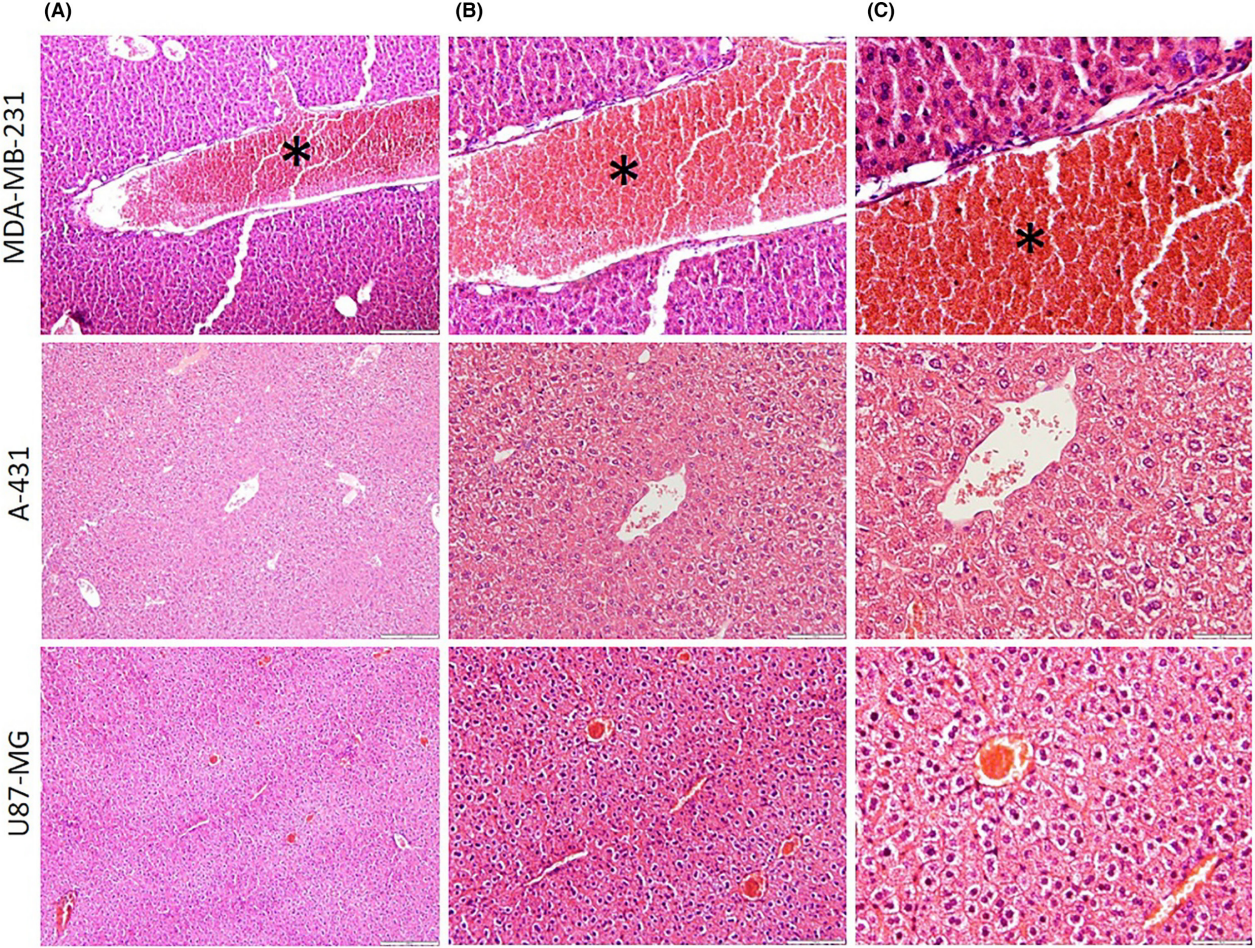
Histopathology analysis using haematoxylin and eosin staining. Panel C (×400) shows higher magnification of photomicrographs in panel B (×200) and panel B is higher than panel A (×100). Stars indicate hyperemia. No metastatic foci were seen.

**FIGURE 6:**
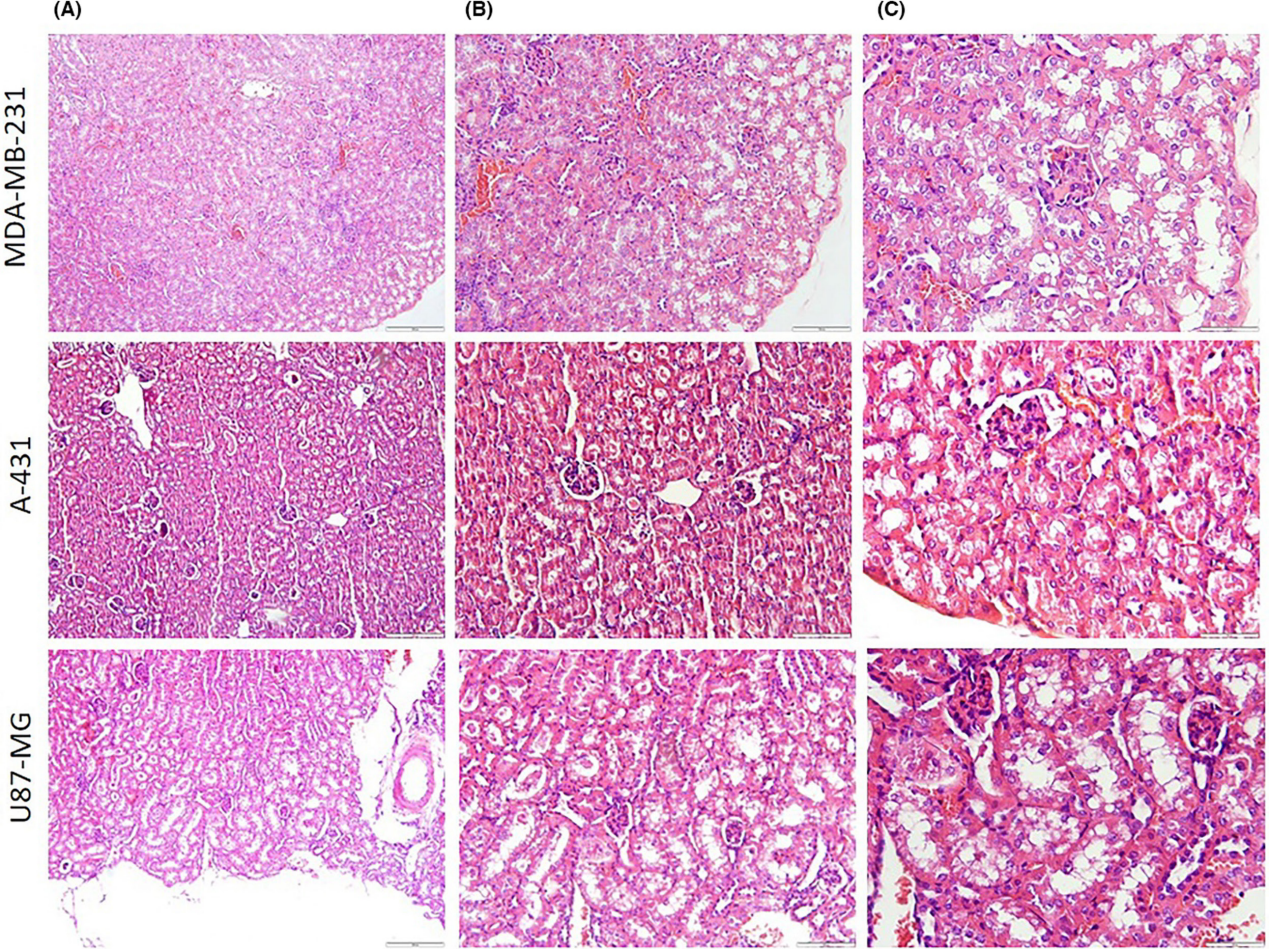
Histopathological analysis of kidney tissues (haematoxylin and eosin staining). Panel C (×400) shows higher magnification of photomicrographs in panel B (×200) and panel B is higher than panel A (×100). All groups showed the normal renal structure and no metastatic foci were seen.

No metastases were found in the liver and kidney using microscopic histopathologic evaluation in all the 12 groups (Figures 5 and 6).

## 4 DISCUSSION

Anti‐cancer drugs are ussualy evaluated on animal xenograft models after in vitro studies.^13^ The immunocompromised mice models, such as Nude (nu), severe‐combined immunodeficient (SCID) and nude SCID gamma (NSG) strains, are widely used as hosts for cancer cell lines derived from human tumours. However, nude mice have significant disadvantages, including high cost, high mortality rate, high sensitivity, low tumorigenesis rate and difficult maintenance.^14^

All anti‐cancer agents that have received clinical approval have been assessed using traditional preclinical research models. The process of conducting xenograft studies can be extremely complicated, starting with the choice of the best animal model, selection of the tumorigenic cell line, administration strategy, dosing and tumour growth rate analysis.^15^, ^16^ To increase rates of tumour engraftment, ionizing irradiation to the whole body can also cause a more severe immune deficiency in mice.^17^ The immune system can be ablated by a radiation dose of 4–6 Gy in athymic nude mice, while 2–3 Gy can be used on NSG mice (which are already radiation sensitive).^13^ However, these procedures are very costly and have a high mortality rate.

Numerous immunosuppressive drugs, including CsA, tacrolimus, ketoconazole, CP, azathioprine, glucocorticoids and methotrexate, are used alone or in combination to prevent the rejection of human tumour xenografts in murine models.^1^, ^5^, ^18^ CsA, a potent immunosuppressive drug, was first used to induce tumour xenograft development in C3H mice.^18^ CsA selectively inhibits calcineurin; thereby, interleukin 2 and other cytokine transcription in T lymphocytes is impaired. Since more than 30 years ago, calcineurin inhibitors have been the cornerstone of immunosuppression in solid organ transplants.^19^

There are a number of reports on immune system suppression using chemical compounds as a single agent or in a combination. Fayzullin et al. used a combination of CsA (30 mg/kg) and ketoconazole (10 mg/kg) to induce immunosuppression in BALB/c mice.^20^ Iranpour et al. suppressed the immune system of male C57BL/6 mice by combination of CsA, itraconazole and co‐amoxiclav.^21^ Cunha et al. reported xenotransplantation (cross‐species transplantation) of human cells of glioblastoma in rats by CsA treatment (5 mg/kg, daily) until the final step of experiments.^8^ Du et al. used CsA (10 mg/kg, daily) to develop xenografts of human hepatocarcinoma cells in C57BL/6 mice.^22^ According to the report of Rose et al., CsA (2–3 mg/kg) in combination with ketoconazole (10 mg/kg) was effective for the successful transplantation of xenologous skin in an immunosuppressed sheep model.^23^ Akhter et al. reported successful induction of human colon adenocarcinoma cells in rats using daily CsA administration.^6^

In this study, CsA, ketoconazole and CP drugs were used to improve an immunosuppression protocol in order to induct human tumour xenografts in mice. Ketoconazole inhibits fungal cytochrome P450 and blocks the production of ergosterol from lanosterol.^24^ Ketoconazole helps CsA circulation and prevents potential fungal infection, which is frequently associated with CsA treatment. The alkylating agent CP prevents DNA replication. In addition, it significantly it reduces the number of natural killer cells, B‐ and T‐cells, and neutrophils.^5^, ^25^

A significant reduction in the total WBC and lymphocyte counts in mice receiving CsA and ketoconazole indicates that immune is significantly suppressed. Metastatic potential studies using histopathology revealed metastatic cases neither in liver tissues nor in kidney tissues.

Thus, using a multimodal treatment with three drugs including CsA, ketoconazole and CP seems to be effective in inducing human tumours of the cells (MDA‐MB‐231, A‐431 and U‐87‐MG) cells in BALB/c. All three types of xenografts kept growing tumour volume for 8 weeks after tumour induction.

## AUTHOR CONTRIBUTIONS

**Abolfazl Amini:** Conceptualization (equal); data curation (equal); formal analysis (equal); funding acquisition (equal); investigation (equal); methodology (equal); project administration (equal); resources (equal); software (equal); validation (equal); writing – original draft (equal); writing – review and editing (equal). **Gholamreza Mesbah:** Data curation (equal); investigation (equal); methodology (equal); resources (equal); validation (equal); visualization (equal); writing – original draft (equal); writing – review and editing (equal). **Fatemeh Tash Shamsabadi:** Data curation (equal); formal analysis (equal); validation (equal); writing – review and editing (equal). **Mohammad Ali Zeyghami:** Conceptualization (equal); data curation (equal); formal analysis (equal); methodology (equal); validation (equal); visualization (equal); writing – review and editing (equal). **Yaghoub Safdari:** Supervision (equal); writing – review and editing (equal).

## FUNDING INFORMATION

This project was supported by Golestan University of Medical Sciences (grant number: 110121).

## CONFLICT OF INTEREST STATEMENT

The authors confirm that there are no conflicts of interests.

## CONSENT FOR PUBLICATION

Not applicable.

## Data Availability

No dataset was generated during this study.
